# Vitiligo: From Pathogenesis to Treatment

**DOI:** 10.3390/jcm13175225

**Published:** 2024-09-03

**Authors:** Reinhart Speeckaert, Elise Van Caelenberg, Arno Belpaire, Marijn M. Speeckaert, Nanja van Geel

**Affiliations:** 1 Department of Dermatology, Ghent University Hospital, 9000 Ghent, Belgium; elise.vancaelenberg@ugent.be (E.V.C.); arno.belpaire@ugent.be (A.B.); nanja.vangeel@ugent.be (N.v.G.); 2 Department of Nephrology, Ghent University Hospital, 9000 Ghent, Belgium; marijn.speeckaert@ugent.be

**Keywords:** vitiligo, non-segmental, segmental, melanocytes, JAK, IFN-γ

## Abstract

Recent advances in vitiligo have provided promising treatment options, particularly through understanding the immune-mediated mechanisms leading to depigmentation. The inflammatory components in both vitiligo (non-segmental) and segmental vitiligo have similarities. Both are believed to result from an immune-based destruction of melanocytes by anti-melanocyte-specific cytotoxic T cells. The JAK-STAT pathway is activated with IFN-γ as the crucial cytokine and Th1-associated chemokines such as CXCL9 and CXCL10 recruit immune cells towards vitiligo skin. Nonetheless, clear differences are also present, such as the localized nature of segmental vitiligo, likely due to somatic mosaicism and increased presence of poliosis. The differing prevalence of poliosis suggests that the follicular immune privilege, which is known to involve immune checkpoints, may be more important in vitiligo (non-segmental). Immunomodulatory therapies, especially those targeting the JAK-IFNγ pathway, are currently at the forefront, offering effective inhibition of melanocyte destruction by cytotoxic T cells. Although Janus Kinase (JAK) inhibitors demonstrate high repigmentation rates, optimal results can take several months to years. The influence of environmental UV exposure on repigmentation in patients receiving immunomodulating drugs remains largely underexplored. Nonetheless, the combined effect of phototherapy with JAK inhibitors is impressive and suggests a targeted immune-based treatment may still require additional stimulation of melanocytes for repigmentation. Identifying alternative melanocyte stimulants beyond UV light remains crucial for the future management of vitiligo.

## 1. Introduction

Due to its status as the most frequent depigmentation disorder, a lot of effort has been undertaken to elucidate the pathogenesis of vitiligo. The progress in unraveling the immunological mechanisms leading to the destruction of melanocytes has contributed to the development of new therapeutic options. However, a clear distinction should be made between vitiligo (non-segmental) and segmental vitiligo. While the immunological mechanism between both forms of vitiligo largely overlaps, the main cause of segmental vitiligo is likely somatic mosaicism, which causes a shorter duration of the inflammatory phase and different treatment options (e.g., pigment cell transplantation) [1]. Previously, other hypotheses such as oxidative stress, neural mechanisms, melanocytorrhagy, melanocyte senescence and the self-destruct theory have been put forward but gained less attention in the last decade. Interesting findings that link inflammation to other factors (e.g., oxidative stress, genetics, neuropeptides) have been published [2,3]. This strengthens the convergence theory that multiple pathways interact in the development of vitiligo, although the autoimmune mechanisms are currently considered to be most crucial [4]. Better insights in the pathways leading to the differentiation and migration of melanocytes and melanocyte stem cells are required. This is evident from the limited therapeutic approaches focusing on repigmentation by activating melanocytes.

### 1.1. Pathogenesis

#### 1.1.1. Vitiligo (Non-Segmental)

Cytotoxic T cells play a significant role in vitiligo by targeting epidermal melanocytes. However, these cells are also found in ‘healthy’ individuals and are not exclusively associated with vitiligo [5]. In normal conditions, these antigen-specific T cells exhibit only a moderate recognition capacity and become anergic [5]. In vitiligo patients, this balance is shifted, leading to an increased activation of these cytotoxic T cells. Immunotherapies against melanoma have pointed to immune checkpoints as crucial regulators of peripheral tolerance against melanocytes. Vitiligo-like lesions have been observed during anti-Programmed death-ligand 1 (PD-L1) treatment (pembrolizumab) in up to 25% of melanoma patients and are associated with improved survival rates [6]. This suggests that vitiligo represents an overactive immune protection against melanoma, leading to collateral damage of healthy melanocytes. Although some clinical signs (e.g., confetti-like depigmentation and lack of acrofacial distribution) have been suggested to be more prevalent in melanoma-associated leukoderma, blinded experts in the field were not able to distinguish vitiligo from melanoma-associated depigmentation based on the clinical presentation [7,8,9]. While melanoma represents immune tolerance, vitiligo can be considered as the other side of the spectrum with an excessive inflammatory response. From a health perspective, the optimal immune response would be an attack restricted against melanocytes that have gained the potential to become malignant and reduce the development of melanoma. Vitiligo patients carry a decreased risk of melanoma and non-melanoma skin cancer: Teulings et al. found a 3-fold lower probability of developing non-melanoma skin cancer (NMSC) [adjusted odds ratio (OR) = 0.28; 95% confidence interval (CI) = 0.16–0.50] and melanoma [adjusted OR = 0.32; 95% CI = 0.12–0.88] in vitiligo patients in the Netherlands [10]. This was confirmed in the UK, where a 38% reduced risk of skin cancer was found [melanoma: adjusted hazard ratio (aHR): 0.39 (95% CI = 0.23–0.65); squamous cell carcinoma: aHR 0.67 (95% CI = 0.49–0.90); basal cell carcinoma: aHR 0.65 (95% CI = 0.51–0.83)] [11]. In addition, further supporting evidence from a meta-analysis of nine studies confirmed the decreased risk of keratinocyte cancer in vitiligo patients [12]. This protective effect could be attributed to several mechanisms. The increased immunosurveillance with decreased negative feedback regulation facilitates the activity of cytotoxic T cells which may also induce keratinocyte destruction. A bystander killing of keratinocytes during the active melanocyte-specific attack has been proposed as an additional explanation [12]. In that regard, it should be noted that melanocytes are also found in basal cell carcinomas, albeit in a higher degree compared to squamous cell carcinoma [13]. Several non-immune processes could also play a role, including factors involved in cell apoptosis such as TP53 and microRNAs associated with cell survival [12]. An inverse genetic relationship exists between skin cancer risk and vitiligo. Several genetic variants in Tyr, MC1R-DEF8 and RALY-EIF2S2-ASIP-AHCY-ITCH loci correlate with an increased risk of melanoma, basal cell carcinoma and squamous cell carcinoma but are also linked to a decreased risk of vitiligo [14]. The treatment of vitiligo therefore involves navigating the delicate balance of a propensity to autoimmunity and beneficial aspects of a strong immunosurveillance such as a good antitumoral and antiviral protection towards an optimal immune response. Overtreatment of the immunological component should be avoided, as excessive treatment is likely to result in increased cancer risk and viral infections [15].

##### Melanocyte-Specific T Cells

**Figure 1:**
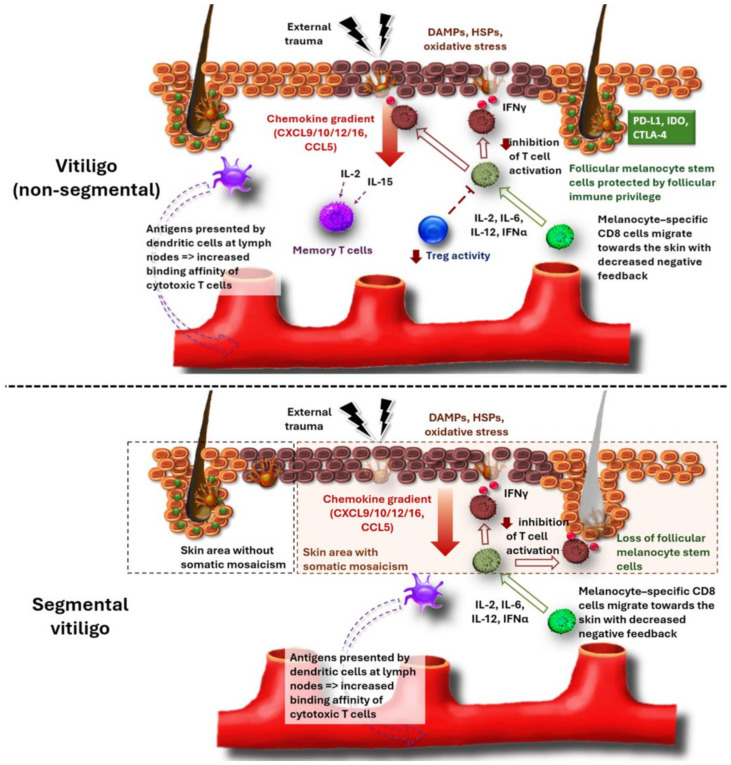
Pathogenesis of vitiligo (non-segmental) and segmental vitiligo. In vitiligo (non-segmental), external trauma induces DAMPs, HSPs and oxidative stress in keratinocytes and melanocytes, resulting in chemokine production. CXCL9/10/12/16 and CCL5 recruit cytotoxic T cells to the skin, which are less restrained when attacking melanocytes due to impaired Treg activity. Antigens of apoptotic melanocytes are taken up by antigen-presenting cells, increasing production of anti-melanocytic T cells. Memory T cells settle into lesional skin, limiting repigmentation and inducing disease relapses.

In vitiligo, melanocyte-specific T cells are less restrained by regulatory T cells (Tregs) and immune checkpoints and migrate towards the skin via a chemokine gradient. Although the initial steps driving this chemokine production are not fully elucidated, danger-associated molecular patterns (DAMPs) and oxidative stress seem crucial (Figure 1). Both can be induced by external trauma leading to the clinically visible Koebner’s phenomenon. Indeed, several DAMPs [e.g., high-mobility group box 1 (HMGB1), S100B, heat-shock protein 70 (HSP70)] are increased in progressive vitiligo [16,17,18]. Additionally, during melanogenesis, high levels of oxidative stress are produced. Consequently, oxidative stress may take a more central place in the development of vitiligo compared to other skin disorders. Factors such as UV radiation, external trauma and inflammation can all increase the release of reactive oxygen species (ROS) by melanocytes and keratinocytes [19]. In response, the stressed keratinocytes and melanocytes produce chemokines. C-X-C motif chemokine ligand (CXCL)9, CXCL10, CXCL12, CXCL15, CXCL16 and Regulated on Activation, Normal T cell-Expressed and -Secreted (RANTES), which are elevated during disease flares, are linked to a T helper 1 response and the recruitment of cytotoxic T lymphocytes [20,21,22]. Furthermore, immune cells are activated by cytokines such as IL-6, IL-12 and IFN-α. A significant breakthrough has been the discovery of the crucial IFN-γ production by cytotoxic T cells, which is a key factor in melanocyte destruction in vitiligo [23]. During disease flares, the affinity for melanocyte antigens further increases, leading to a self-propagating inflammatory response. Several melanocyte antigens have been identified, including MelanA/Mart1, gp100 and tyrosinase [24]. Melanocyte-specific CD8 cells produce IFN-γ and apoptosis-inducing factors such as perforin and granzyme leading to melanocyte destruction [25,26]. Additionally, direct cell–cell interaction via Fas-Fas ligand signaling leads to the activation of caspases, resulting in melanocyte destruction [27]. CXCL10 stimulates CXCR3B expression on melanocytes, also inducing melanocyte apoptosis. The residual melanocytes contain co-stimulatory receptors in response to IFN-γ that stimulate T-cell proliferation and activation, hence augmenting the anti-melanocytic response [28]. Vitiligo melanocytes may be less capable of expressing immune checkpoints such as PD-L1 compared to ‘healthy’ melanocytes, reducing their capacity to prevent immune-based destruction [29]. PD-L1 expressed by melanocytes binds to PD-1 on cytotoxic T cells, leading to T cell anergy and immune tolerance to self-antigens. This mechanism is crucial for preventing autoimmunity. If PD-L1 expression is reduced or therapeutically inhibited [e.g., by anti-PD-1 treatment (pembrolizumab) against melanoma], cytotoxic T cells remain activated and are not restrained from attacking melanocytes [30]. After the disappearance of melanocytes, it takes about 4–6 weeks before clinical depigmentation is apparent due to the gradual epidermal turnover [31]. Historically, a neuroendocrine hypothesis has also been put forward for the pathogenesis of vitiligo. Most research suggests that a local accumulation of neuropeptides (e.g., substance P, neuropeptide Y) is indeed present in active vitiligo skin [32,33]. However, these neuropeptides are produced by dermal nerves, most likely as a consequence of inflammation, although regulation by the central nervous system cannot be completely excluded [4].

##### Melanocyte Reservoirs

In the early phases of non-segmental vitiligo, hair pigmentation is usually spared. This immune privilege of the hair follicles is essential for future repigmentation, as melanocyte stem cells are primarily located in the hair bulge [34]. Additional melanocyte reservoirs of amelanotic melanocytes are present in the outer root sheath and the hair bulb. Other locations of melanocyte stem cells are the basal membrane of the interfollicular epidermis and the secretory part of the eccrine sweat glands [35].

##### Memory T Cells

After melanocyte destruction, melanocytic antigens are presented at the lymph nodes, increasing the binding affinity of cytotoxic T cells. Subsequently, memory T cells settle into lesional skin, inducing long-term depigmentation and disease relapses [36]. IL-15 and IL-2 are involved in the development and maintenance of skin memory T cells in vitiligo. IL-2 is mainly produced by T helper cells, dendritic cells and, to a lesser extent, by cytotoxic T cells [37]. IL-15 is produced by Langerhans cells, dendritic cells, keratinocytes and other fibroblasts [38].

NKG2D expression on memory CD8+ T cells indicates an activated status [39]. Repigmentation is substantially higher in cases where the inflammatory response is well controlled. Nonetheless, additional triggers besides anti-inflammatory treatment are, in most cases, required to allow differentiation and migration of melanocyte precursors. UV exposure remains the gold standard for activating melanocytes. Wnt-signaling and Prostaglandin E2 (PGE2) are also linked to melanocyte stimulation [40].

##### Memory Tregs

Tissue resident memory Tregs and antigen-specific Tregs are reduced in lesional and perilesional vitiligo skin [41]. CCR6 has been identified as a chemokine receptor crucial for Treg migration into vitiligo skin [42]. Absence of CCL20-CCR6 signaling leads to a reduced suppression of the activity and proliferation of CD4 and CD8 tissue resident memory T cells. CCR5 expression is not required for Treg recruitment but is important for the suppressive function of Tregs by properly positioning Tregs in the skin [43]. In a mouse model, overexpression of CCL22, which binds to CCR4, resulted in decreased depigmentations. CCL22 activates the migration and activity of Tregs in vitiligo, which has been shown to be decreased in vitiligo skin, whereas no changes were found for CCR4, CCR5, CCR8 and cutaneous lymphocyte antigen (CLA) [44,45]. CCR7 and its ligand CCL21 are also involved in Treg migration and CCL21 is significantly decreased in vitiligo skin [46]. A meta-analysis of 30 studies found decreased numbers of Tregs in vitiligo patients [47]. FOXP3, a key transcription factor of Tregs, is also reduced in the blood and skin of vitiligo patients, which coincides with a decreased Treg-mediated suppression of cytotoxic T cells [47]. In vitiligo patients, regular Tregs have a higher tendency to transform into Th1-like T-bet^+^IFN-γ^+^Tregs, which exhibit a decreased capacity to suppress the proliferation and activation of cytotoxic T cells [48]. Interestingly, NB-UVB and Treg-specific treatments increase Treg frequency [47].

#### 1.1.2. Segmental Vitiligo

**Figure 2:**
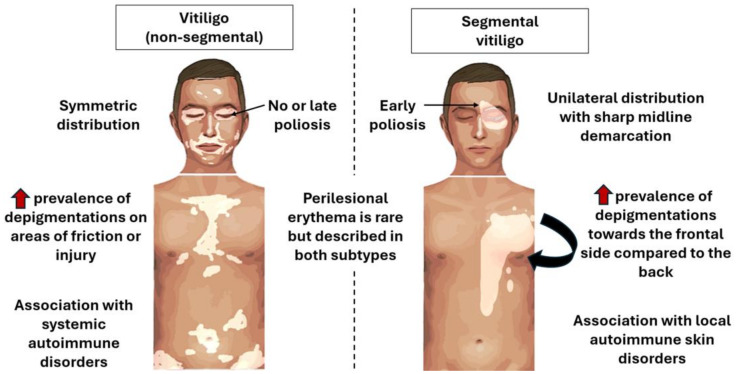
Clinical presentations of vitiligo (non-segmental) and segmental vitiligo. Segmental vitiligo differs from its non-segmental counterpart by a unilateral (black arrow) instead of symmetric distribution, early poliosis compared to no or late poliosis, increased risk of local auto-immune skin disorders instead of systemic autoimmune diseases (red arrows) and typical distribution patterns not corresponding to the predilection areas characteristic for vitiligo (non-segmental).

Segmental vitiligo has a unique presentation, with skin depigmentations occurring only on one side of the body with a relatively strict demarcation at the midline (Figure 2). Other major differences compared to vitiligo (non-segmental) are predominant in the age range between 8 and 12 years and a spontaneous end to progression is usually noticed after 1–2 years [49]. Remarkably, poliosis often occurs at an early stage, suggesting that the hair immune privilege and immune checkpoints are less involved in the pathogenesis of segmental vitiligo compared to its non-segmental counterpart [49]. Additionally, segmental vitiligo is less associated with other autoimmune disorders [50]. This is due to the different pathogenic events leading to segmental vitiligo. Although not formally proven, somatic mosaicism of a subgroup of melanocytes is the most likely underlying mechanism. During embryogenesis, melanoblasts migrate and proliferate from the back of the embryo over the sides of the body towards the frontal midline. This unique way to populate the skin with melanocytes is reflected in Blashko’s lines, which are observed in congenital pigmentary disorders [51]. During this phenomenon, mutations in melanocytes are likely given the large numbers of cell proliferations. Consequently, melanoblasts that have migrated towards the frontal midline are more likely to have acquired mutations aligning with the more frequent lesion distribution closer to the frontal midline of the body compared to the back in segmental vitiligo. Additionally, the distribution pattern of segmental vitiligo closely resembles segmental lentiginosis, which is another pigmentary disorder likely caused by somatic mosaicism of melanocytes [52]. At some point in life, an inflammatory response may develop against these genetically different melanocytes. As a result, similarly to vitiligo (non-segmental), melanocyte-specific T cells have also been identified in perilesional segmental vitiligo skin [53]. Serum CXCL10 and IFN-γ are also increased, further strengthening the evidence of the immunological component of segmental vitiligo (Figure 1) [54]. In fact, the pathogenesis may be comparable to other mosaic skin immune disorders such as lichen striatus [55].

Segmental vitiligo also exhibits an innate immune component and increased C-X-C Motif Chemokine Receptor 3 (CXCR3)B mRNA expression both in lesional and non-lesional skin [56]. However, increased CXCR3B-positive melanocytes were only found in lesional but not in non-lesional skin of segmental vitiligo patients compared to healthy controls [56]. This may explain why melanocytes are targeted for immune-based destruction. Increased expression of heat-shock protein (HSP)70 and CXCL16 was reported at the lesional side, suggesting a local susceptibility for vitiligo, which supports the theory of somatic mosaicism [18,21,22]. However, these factors are mainly produced by keratinocytes and not melanocytes. In contrast to vitiligo (non-segmental), segmental vitiligo is less associated with systemic autoimmune disorders, although coinciding localized autoimmune skin diseases (e.g., morphea) can occur [50]. Different expressions of key proteins outside lesional skin may explain the predisposition of segmental vitiligo patients to progress to the non-segmental form. Segmental vitiligo patients have an approximately 10-fold higher risk of developing widespread depigmentations on other body parts [=vitiligo (non-segmental)] compared to healthy controls [56].

## 2. Treatments

### 2.1. Topical Treatments

#### 2.1.1. Topical Treatments Targeting the Immune Response

**Figure 3:**
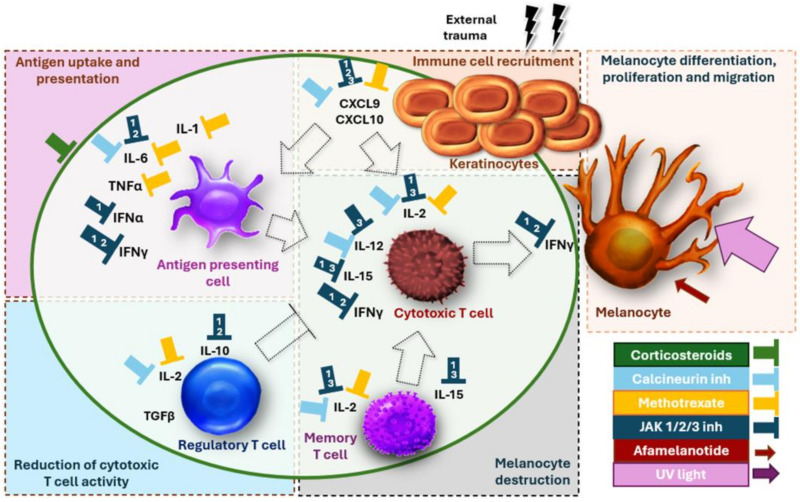
Working mechanisms of different treatments (green circle = inhibited by corticosteroids; 1 = anti-JAK1; 2 = anti-JAK2; 3 = anti-JAK3). Corticosteroids have a broad working mechanism inhibiting cytokines in the early and late phases of the immune response. Calcineurin inhibitors inhibit IL-1, IL-2, IL-6, TNF-α and CXCL9/10. This provides diverse anti-inflammatory effects, reducing immune cell recruitment, antigen presentation and cytotoxic T cell activity. However, the effects of calcineurin inhibitors on IFN-γ production are indirect. JAK1-2 blockers inhibit several driving and effector cytokines, in particular IFN-γ. Their inhibiting effect on IL-10 might reduce regulatory T cell activity, which is less desirable. Anti-JAK3 does inhibit IFN-γ directly but reduces cytotoxic T cell and possibly also memory T cell responses by inhibiting IL-2 and IL-15. Therapies that induce melanocyte proliferation and migration are limited and consist of phototherapy and afamelanotide.

The main topical treatments include corticosteroids, calcineurin inhibitors and JAK inhibitors (Figure 3) [1]. Corticosteroids decrease a broad spectrum of cytokines, chemokines and other factors that reduce T cell activity [57]. Tacrolimus and pimecrolimus inhibit the enzyme calcineurin. This reduces the activity of T cells and decreases the production of IL-2, IFN-γ and TNF-α [58]. Tacrolimus can reduce the chemokines CXCL10 and RANTES [58,59]. Both tacrolimus and pimecrolimus increase melanogenesis and stimulate the migration of melanocytes in vitro [60,61]. Ruxolitinib is a JAK1 inhibitor that inhibits IFN-γ, IL-2, IL-15 and chemokines CXCL9/10/11 and RANTES. Nonetheless, JAK signaling is dispensable for the maintenance of memory T cells, suggesting the need for long-term treatment [62]. Prostaglandins shift the immune system from a Th1 response to a Th2 response. PGE2 has been identified as a key factor in the repigmentation of phototherapy. PGE2 can be applied topically or injected intradermally, with limited data showing improved repigmentation [63]. Prostaglandin F2 shows similar results [64]. Although promising in mice models, topical or systemic simvastatin is not effective in human trials. This is likely because of the lower tolerable dose in humans versus mice, leading to rhabdomyolysis [65,66,67]. Interestingly, some cases with improvement using crisaborole ointment have been reported [68,69]. Roflumilast, another phosphodiesterase-4 (PDE4) inhibitor, decreases oxidative stress in melanocytes and slightly enhances melanogenesis [70]. Topical anti-inflammatory treatments are, in most cases, effective at preventing new lesions or enlarging existing vitiligo lesions. In monotherapy, repigmentation is, however, slow and often mostly visible on the face.

#### 2.1.2. (Topical) Treatments Stimulating Melanocytes

Phototherapy (NB-UVB or excimer laser) has an immunomodulating effect, although its major working mechanism is to stimulate the migration and proliferation of melanocytes and melanocyte precursors and to enhance melanogenesis. Some irritative or slightly damaging treatments such as 5-fluorouracil, fractional ablative laser and microneedling have been administered to induce repigmentation, especially in stable but treatment-resistant patients. Several studies using these treatments have reported increased repigmentation, possibly due to enhanced production of chemokines such as CXCL12 by keratinocytes, melanocytes and dermal fibroblasts. CXCL12 promotes the chemotactic migration of melanocytes by binding to CXCR4 and CXCR7 receptors on melanocytes [71,72,73]. Nonetheless, the plausible risk of Koebner’s phenomenon and lack of large randomized trials limit their widespread adoption. Treatments stimulating melanocyte differentiation, proliferation and/or migration are best combined with anti-inflammatory treatments to improve the chance of repigmentation.

### 2.2. Systemic Treatments

#### 2.2.1. Systemic Treatments Targeting the Immune Response

Mini-pulse corticosteroids have been proposed as a systemic treatment to halt disease progression due to their ability to reduce a wide range of proinflammatory factors and decrease the activity of dendritic cells and T lymphocytes. However, despite these benefits, the repigmentation achieved with corticosteroids is often modest, largely because they do not have a substantial effect on melanocyte stimulation. In contrast, JAK inhibitors have emerged as a promising alternative. By targeting JAK1 and JAK2, these inhibitors effectively block IFN-γ, the key cytokine responsible for melanocyte destruction. Additionally, JAK1 inhibits IL-2, IL-6, IL-15 and IFN-α, while JAK2 reduces IL-12, a key driver of the Th1 pathway. JAK3, on the other hand, inhibits IL-2 and IL-5 (Figure 3).

The immunomodulatory effects of the JAK3 inhibitor ritlecitinib have been thoroughly investigated in a phase II trial, where markers linked to T cell and NK cell activation (CCR7, IL-2 and IL2-RA) were dose-dependently downregulated [74]. Interestingly, soluble IL2-RA has been consistently associated with vitiligo activity, further confirming the on-target efficacy of JAK3 inhibition [75]. Moreover, cytokines like IFN-γ, Th1 markers such as CXCR3 and chemokines like CXCL9 and CCL5 were similarly reduced following treatment. Even though the activity of the Th2 pathway (IL-13, CCL13, CCL18, CCR4) was also reduced, this is likely an off-target effect as the Th2 pathway is not considered central to the pathogenesis of vitiligo [74]. Notably, IL-17A levels remained unchanged following treatment with ritlecitinib, aligning with previous findings that IL-17A inhibition does not offer significant benefit for vitiligo treatment [76].

Conventional immunosuppressants such as methotrexate, cyclosporine and azathioprine may still have a role, especially when JAK inhibitors are contraindicated. Recent studies have revealed that methotrexate, traditionally thought to work via folate metabolism, is actually a JAK1/2 inhibitor, which accounts for its efficacy [77]. Although largely based on retrospective clinical data, low-dose methotrexate has been shown to reduce disease activity and enhance repigmentation when combined with phototherapy [78].

The biologics currently available for psoriasis and atopic dermatitis, including anti-IL-4, anti-IL-13, anti-IL-17, anti-IL-23 and anti-TNF-α treatments, appear to offer limited benefit for vitiligo patients. In fact, no clear data support their efficacy in vitiligo, and several reports have suggested that these biologics may even trigger vitiligo by negatively impacting the immune balance toward the Th1 pathway [79]. The effects of rituximab, an anti-CD20 biologic targeting B lymphocytes, have not been sufficiently explored [80]. Besides biologics, mixed findings have been reported regarding the PDE4 inhibitor apremilast, with outcomes ranging from no significant effect to a modest halt in disease progression and repigmentation [81].

For decades, the value of antioxidants in vitiligo management has been debated. While the general consensus leans toward supporting their use, there are a lack of clinical trials that reproducibly demonstrate enhanced repigmentation or stabilization of disease progression with individual compounds. Moreover, certain antioxidants, like polypodium leucotomos, may be recommended due to their ability to reduce the minimal erythematous dose (MED), thereby decreasing the risk of skin burns following UV exposure.

Overall, oral immune-modulating drugs are effective in halting disease progression, although repigmentation is often slow without combination therapies to stimulate melanocytes. Oral mini-pulse (OMP) dexamethasone administered to progressing vitiligo patients showed arrest of disease activity in 92% of patients [82]. Nonetheless, a meta-analysis showed that >75% repigmentation was only observed in 0–32% of patients when OMP therapy was administered as monotherapy, which was comparable to other treatments [83]. Consequently, topical treatments combined with phototherapy remain the standard for repigmentation according to the latest international treatment guidelines, with oral immunomodulating treatments classified as ‘optional’ [1].

#### 2.2.2. Systemic Treatments Stimulating Melanocytes

Afamelanotide, an α-melanocyte-stimulating hormone (MSH) analog, occupies a rather unique place in the treatment arsenal, being one of the only treatments that stimulates melanocytes. Especially in dark skin types, enhanced repigmentation has been documented in combination with NB-UVB treatment [84,85]. However, the general darkening of the skin induced by afamelanotide accentuates the contrast between vitiligo lesions and healthy skin. Although limited to the treatment period with afamelanotide, this leads to a worse cosmetic appearance, complicating its use [86].

## 3. Future Treatment Options

Future treatment options focus on various strategies to enhance Treg function, which may balance the Th1 pathway. For example, low-dose IL-2 or IL-2 mutein specifically designed to activate Tregs are currently tested in phase II [87]. Additionally, stimulation of CCL22 and CCR6 represent other future targets for enhancing the activity of Tregs [42,44].

The main downside of targeting IFN-γ is the possible systemic adverse events, such as cancer and viral infections. Consequently, a location-specific treatment inhibiting IFN-γ only in the epidermis may be a more appealing approach. In support of this idea, bispecific antibodies have already been successfully tested in mouse models of vitiligo. These antibodies may have two targets; one disease-specific (e.g., IFN-γ), and one skin-specific (e.g., anti-desmoglein) leading to skin retention of the biologic and faster elimination from the blood reducing systemic effects [88].

Meanwhile, immune checkpoints [PD-L1, indoleamine (IDO), cytotoxic T-lymphocyte-associated protein 4 (CTLA-4)] are also currently being explored in drug development [30]. Unlike in cancer immunotherapy, immune checkpoint agonists instead of antagonists are being used. Nonetheless, it remains unclear whether this approach might induce a systemic decrease in immune activity, potentially leading to tolerance for pathogens and malignant cells. Similarly to IFN-γ inhibition, localized options have been proposed. Melanocyte-targeted bispecific PD-1 agonists may only upregulate immune checkpoints around melanocytes, providing an immune-privileged environment while reducing systemic effects [89].

Another major challenge remains the elimination of tissue-resident memory T cells to avoid disease recurrence and treatment-resistant lesions. Ordekisumab (anti-IL15) and auremolimab (anti-IL15Rβ) (CD122) are specific for IL-15 signaling, which is crucial for the maintenance of the IFN-γ linked skin memory response [90]. Finally, many other therapeutic options and drug targets have been put forward, including probiotics, mutant heat-shock proteins, inhibition of DAMPs, prevention of melanocyte apoptosis by inhibiting CXCR3B, anti-NKG2D (targeting CD8/NK cells) and WNT agonists/GSK3b inhibitors (stimulating melanocyte stem cells) [91].

## 4. Conclusions

The evolving landscape of vitiligo underscores a dynamic interplay between innovative therapeutic strategies and a deepening understanding of the disease’s pathophysiology. The identification of the JAK-IFNγ pathway as a pivotal target in the modulation of immune responses offers a promising avenue for effective management. However, the complexities of vitiligo demand a cautious approach, advocating for the development of complementary therapies that ensure both efficacy and safety over prolonged treatment periods. Continued research into alternative melanocyte stimulants will be crucial in refining treatment protocols that are both personalized and comprehensive.
